# Development and validation of new glomerular filtration rate predicting models for Chinese patients with type 2 diabetes

**DOI:** 10.1186/s12967-015-0674-y

**Published:** 2015-09-28

**Authors:** Jinxia Chen, Hua Tang, Hui Huang, Linsheng Lv, Yanni Wang, Xun Liu, Tanqi Lou

**Affiliations:** Division of Nephrology, Department of Internal Medicine, The Third Affiliated Hospital of Sun Yat-sen University, Guangzhou, 510630 China; Institute of Nephrology, Guangdong Medical College, Zhanjiang, Guangdong China; Department of Cardiology, Guangdong Province Key Laboratory of Arrhythmia and Electrophysiology, Sun Yat-Sen Memorial Hospital, Sun Yat-Sen University, Guangzhou, China; Operation Room, The Third Affiliated Hospital of Sun Yat-sen University, Guangzhou, China

**Keywords:** Glomerular filtration rate, Type 2 diabetes, Artificial neural network, Serum creatinine, Body mass index

## Abstract

**Background:**

Previous researches has depicted that the performance of the recommended glomerular filtration rate (GFR)-estimating equations in the type 2 diabetic population is inferior to that in the non-diabetic population. We attempted to develop new GFR-predicting models for use in Chinese patients with type 2 diabetes in this study.

**Methods:**

We enrolled 519 type 2 diabetic patients including a development data-set (n = 276), an internal validation data-set (n = 138) and an external validation data-set (n = 105) to establish new GFR-predicting models. 99mTc-DTPA-GFR revised by the dual sample method was referred to as the gold GFR standard.

**Results:**

Based on sex, age, serum creatinine and new predictor variables [body mass index (BMI), hemoglobinA1C, and urinary albumin creatinine ratio], eight new regression models and eight artificial neural network (ANN) models were developed. In the external validation group, only ANN3 was superior in both precision and accuracy over the original CKD-EPI equation (precision, 20.5 vs. 24.2 mL/min/1.73 m^2^, *P* < 0.001; 30 % accuracy, 88.6 vs. 80.6 %, *P* = 0.02).

**Conclusions:**

ANN3 based on sex, age, serum creatinine and BMI is the optimal model for GFR estimation in Chinese patients with type 2 diabetes.

**Electronic supplementary material:**

The online version of this article (doi:10.1186/s12967-015-0674-y) contains supplementary material, which is available to authorized users.

## Background

An accurate assessment of glomerular filtration rate (GFR) is crucial for correct drug dosing, as well as for detecting, managing, and predicting the prognosis of chronic kidney disease (CKD) in diabetes [1]. However, analysis has indicated that the Cockcroft–Gault (CG) equation [2], the modification of diet in renal disease (MDRD) equation [3] and the Chronic Kidney Disease Epidemiology Collaboration (CKD-EPI) equation [4], which were recommended for the estimation of GFR, are inaccurate in diabetic populations [5–7]. In accordance with the above studies [5–7], our previous result demonstrated that the accuracy of the CKD-EPI equation could be influenced by diabetic status [8].

There are several explanations for the discrepancy between diabetic and non diabetic population. To our knowledge, the establishment of traditional equations was dependent on the regression model in a sizable number of participants without consideration of physiology, thus the performance of traditional equations can be poor when they are applied in a significantly different population. Only a very small percentage of diabetic patients were involved in establishing the CG and MDRD equations, and only 27 % of the subjects in the CKD-EPI equation were diabetic. Further, attention should be paid to the special physiology of type 2 diabetic patients. For example, in the early stage of diabetic nephropathy, owing to hyperfiltration, diabetic patients often represent with high values of GFR [9]. It is well recognized that GFR can be increased by acute hyperglycemia in type 2 diabetes with normal renal function. A study involving 193 diabetic patients by Rigalleau [10] showed that the relationship also existed in more advanced stages of renal impairment, and that a reduction in glycosylated hemoglobin (HbA1c) was related to a reduction in GFR after treatment for several months. Moreover, most individuals with type 2 diabetes were overweight or obese. The fact that the obese people tented to have a higher proportion of fat and a lower content of muscle mass compared to the non-obese people has impacts on GFR [11]. In addition, several studies implied that the level of serum creatinine was remarkably lower in diabetes due to the lower muscle mass volume [9, 12]. Special attention should be paid to albuminuria in diabetes. The appearance of albuminuria initiates the loss of GFR [13] and several reports have identified that the level of albuminuria correlates with the level of the loss of renal function in people with diabetes and obvious renal diseases [14–16]. Babazono [17] stressed that higher levels of urine albumin-to-creatinine ratio (UACR) are associated with a greater rate of decline in eGFR in Japanese diabetic patients. In the supplementary appendix of a study by Lesley, the performance of the CKD-EPI equation varied in different proteinuria subgroups [18].

Equations currently estimate GFR on the basis of GFR determinants as well as non-GFR determinants. GFR determinants are referred to as GFR filtration markers, whereas non-GFR determinants include demographic and clinical variables which influence GFR determinants. The combination of novel filtration markers and non-GFR determinants can contribute to the improvement in performance by reducing the bias caused by non-GFR determinants [1]. Besides, advances in statistical techniques and the development of novel strategies can benefit GFR estimation. An artificial neural network (ANN) as a common mathematical modeling method has been employed extensively in engineering prediction and the application of ANN in medicine and biology is also encouraging. Our previous study, which consisted of 1230 patients with chronic kidney disease, verified that the performance of ANN was equal to traditional regression [19].

Taking the above into account, we aim to develop an optimal GFR-predicting model for Chinese type 2 diabetic patients by applying the traditional regression method as well as an ANN with the addition of new variables [including body mass index (BMI), HbA1c and UACR] as non-GFR determinants.

## Methods

### Subjects and study design

The study recruited consecutive patients who had known type 2 diabetes for whom complete clinical data were available in the Third Affiliated Hospital of Sun Yat-sen University, China. Exclusion criteria were: (1) age less than 18 years old; (2) having acute kidney function deterioration (the level of serum creatinine on the day of undergoing GFR measurement differed more than 15 % compared with that on the day of admission), skeletal muscle atrophy, edema, pleural effusion or ascites, malnutrition, amputation, heart failure, or ketoacidosis; (3) being treated with dialysis at the time of the study; (4) taking cimetidine, trimethoprim and injection of albumin or diuretics intravenously before the measurement of GFR. Finally 519 type 2 diabetic patients were enrolled. Patients treated from January 2005 through December 2012 were randomly divided into the development data-set (n = 276) and the internal data-set (n = 138). Data obtained from January 2013 to June 2013 were used as the external validation data-set (n = 105). Written informed consent was obtained from all subjects. The study was approved by the institutional review board at the Third Affiliated Hospital of Sun Yat-sen University.

### Laboratory measurements

The measurement of GFR was obtained by a technetium 99 m diethylene-triaminepentaacetic acid (^99m^Tc-DTPA) renal dynamic imaging method (modified Gate’s method), using a Millennium TMMPR SPECT with the General Electric Medical System (Discovery VH, GE Healthcare, Little Chalfont, UK). The details have been described previously [20]. The measured GFR (mGFR) was calibrated to equal the dual plasma sample ^99m^Tc-DTPA GFR. Applying Open Epi software (Version 2), the minimum sample size was calculated as 36 [21] (95 % confidence level and 80 % power). The calibrated GFR measurement was referred to as the standard GFR (sGFR) in our study. Two and four hours after the injection of ^99m^Tc-DTPA into the opposite forearm, blood samples were drawn intravenously and collected in heparinized tubes. Radioactivity in the separated plasma was recorded by a multi-function well counter (ZD-6000 multi-function instrument from Zhida Technology Company, Xian, China). Serum creatinine analysis was performed by a Hitachi 7180 autoanalyzer (Hitachi, Tokyo, Japan; reagents from Roche Diagnostics, Mannheim, Germany) using the enzymatic method, and after the year 2010 serum creatinine was traceable by isotope dilution mass spectrometry. HbA1c was detected by high performance liquid chromatography, while UACR was determined by the immuneturbidimetric assay.

### Metrics for the development of the new regression models

The development of the new regression models was based on both the development and internal validation data sets. The predictor variables involved in the establishment were age, sex, serum creatinine, BMI, HbA1c, and UACR. Age, sex, and serum creatinine were included in all new models, with BMI, HbA1c, and UACR added separately or in combination. In the development of the new equations, sGFR and serum creatinine were transformed to a log scale, while BMI, HbA1c, and UACR were on the natural scale. Least squares linear regression was adopted to relate sGFR to the predictor variables. As the method mentioned in the establishment of the CKD-EPI equation, a nonparametric smoothing spline was used to configure the shape of the relationship of log standard GFR with log creatinine, and the nonlinearity relationship represented in the smoothing splines was characterized by means of piecewise linear splines.

### Metrics for the development of the new ANN models

The new ANN models were programmed by MATLAB 2011A (The MathWorks Inc, Natick, MA, USA). A three-layer back-propagation (BP) network consisting of an input layer, a hidden layer and an output layer was established. The predictor variables, the same as the description above, were the input variables with standard GFR as the output variable. Each neuron in the hidden layer took the S function as an exciting function and with different numbers of neurons in the hidden layer (1–11), several networks were programmed. After the random initialization, all net works were trained in the development data-set by learning the rule of back propagation. Their performance in the internal validation data-set determined the optimal network. Performance was assessed by mean square error in the internal validation data-set and the smallest mean square error meant the best performance. With thresholds and weights specified after training, the output of the network was calculated by the weighted summation of each neuron to approximate sGFR. The introduction of the genetic algorithm into the BP network (GABP network) enabled optimized initialization of the weights and thresholds, leading to improvement of performance of the ANN models. In the GABP network, encoded as a chromosome, all weights and thresholds of one network evolved from one generation to another through the progression of mutation and crossing. The initial weights and thresholds were chosen for the next generation if the network demonstrated better performance in the internal validation data-set. Superior initial weights and thresholds were eventually applied in the initialization of the network. Details of the construction of the new ANN model are presented in the Additional file 1.

### Determination of the optimal models

All the new models were compared with the new Japanese equations and the CKD-EPI equation in the external validation data set. The performance was defined by bias, precision and accuracy.

### Expression of the equations

CKD-EPI equation [4]

$${\text{GFR}} = 141 \times {\text{min}}\left( {{\text{Scr}}/\kappa ,1} \right)^\alpha \times {\text{max}}\left( {{\text{Scr}}/\kappa ,1} \right)^{{ - 1.209}} \times 0.993^{{{\text{Age}}}} \times 1.018\;\left( {{\text{if female}}} \right) \times 1.159\;\left( {{\text{if black}}} \right)$$

κ is 0.7 for female and 0.9 for male, α is 0.329 for female and 0.411 for male, min indicates the minimum of SC/κ or 1, and max indicates the maximum of SC/κ or 1Japanese equation 1 [22]

$${\text{GFR}} = 1 9 4 \times {\text{Scr}} ^{- 1.0 9 4} \times {\text{Age}}^{ - 0. 2 8 7} \times 0. 7 3 9\;\left( {\text{if female}} \right)$$

Japanese equation 2 [9]

$${\text{GFR}} = 1 9 4 \times {\text{Scr}} ^{- 1.0 9 4} \times {\text{Age}}^{ - 0. 2 8 7} \times 0. 7 3 9\left( {\text{if female}} \right)/(0. 4 2 8 + 0.00 8 5 \times {\text{HbA1c}})$$

### Statistical analyses

Results are expressed as mean ± SD or as median. Bias was assessed as the median of the difference between sGFR and eGFR, and precision was defined as the inter-quartile range (IQR) of the difference. Accuracy was measured as the percentage of eGFRs within 30 % of the sGFR. The 95 % confidence intervals were calculated by the bootstrap method (2000 bootstraps). The quantitative variables between two data-sets were compared using the independent samples *t* test or the Mann–Whitney test. Differences and accuracy within the data-set were compared by Wilcoxon signed rank test and McNemar test. All analyses were conducted using SPSS software (version 13.0 SPSS), R (R i386 3.0.2) and MATLAB software (version 2011b).

## Results

### Calibration of GFR

To calibrate our measured GFR to the dual plasma samples method, 36 type 2 diabetic subjects [mean age was 63.3 ± 12.3 y (range 38–82) with a mean mGFR of 66.9 ± 30.1 ml/min/1.73 m^2^ (range 16.7–145.6 ml/min/1.73 m^2^)] were selected randomly. The dual plasma samples method 99mTc-DTPA clearance was performed simultaneously with renal dynamic imaging. The ^99m^Tc-DTPA renal dynamic imaging GFR value can be calibrated equally to the dual plasma samples ^99m^Tc-DTPA clearance, using a linear regression equation: dual plasma sample ^99m^Tc-DTPA-GFR (ml/min/1.73 m^2^) = 3.706 + 1.039 × ^99m^Tc-DTPA renal dynamic imaging-GFR (ml/min/1.73 m^2^) (R^2^ = 0.879, *P* < 0.001).

### Clinical characteristics of the development, internal validation data-sets and external validation data-set

Mean sGFR of the development and internal validation data-sets was 80.9 ± 29.0 mL/min/1.73 m^2^. The mean age, mean BMI, mean sGFR, and the distribution of HbA1C of the external validation data set were similar to the development and internal validation data-sets. Clinical characteristics of the diabetic patients in each data-set are provided in Table 1.

**Table 1:** Clinical characteristic of the development, internal validation data sets and external validation data set

	Development and internal validation	External validation	P
N	414	105	
Male/female (%)	54.3/45.7	58.1/41.9	0.49
Age (y) mean ± SD	59.8 ± 13.1	60.2 ± 10.6	0.76
<40 n (%)	29 (7.0)	3 (2.9)	0.26
40–65 n (%)	231 (55.8)	67 (63.8)	
>65 n (%)	154 (37.2)	35 (33.3)	
Diabetes duration (y) median (IQR)	7.0 (8.0)	9.0 (9.0)	0.01
Weight (kg) mean ± SD	66.5 ± 12.3	68.2 ± 12.1	0.22
Height (cm) mean ± SD	162.8 ± 8.6	162.6 ± 7.9	0.81
BSA (m^2^) mean ± SD	1.7 ± 0.2	1.7 ± 0.2	0.40
BMI (kg/m^2^) mean ± SD	25.0 ± 3.5	25.7 ± 3.4	0.07
<20 n (%)	23 (5.6)	1 (1.0)	0.01
20–25 n (%)	189 (45.7)	52 (49.5)	
25–30 n (%)	171 (41.5)	36 (34.3)	
>30 n (%)	30 (7.2)	16 (15.2)	
Serum creatinine (mg/dl) median (IQR)	0.8 (0.6)	0.9 (0.7)	0.34
HbA1C (%) median (IQR)	8.5 (3.4)	8.3 (3.9)	0.83
<6 % n (%)	40 (9.7)	9 (8.6)	0.76
6–8 % n (%)	146 (35.3)	41 (39)	
>8 % n (%)	228 (55.1)	55 (52.4)	
UACR (mg/g) median (IQR)	42.6 (219.0)	62.9 (166.2)	0.01
<30 mg/g n (%)	162 (39.1)	19 (18.1)	<0.001
30–300 mg/g n (%)	144 (34.8)	60 (57.1)	
>300 mg/g n (%)	108 (26.1)	26 (24.8)	
sGFR (ml/min/1.73 m^2^)	80.9 ± 29.0	83.7 ± 25.9	0.40
<15 n (%)	0 (0)	0 (0)	0.43
15–29 n (%)	16 (3.9)	1 (1.0)	
30–59 n (%)	85 (20.5)	19 (18.1)	
60–90 n (%)	154 (37.2)	43 (41.0)	
>90 n (%)	159 (38.4)	42 (40.0)	

*SD* standard deviation, *IQR* interquartile range, *BSA* body surface area, *BMI* body mass index, *UACR* urine albumin creatinine ratio, *sGFR* standard glomerular filtration rate

### Development of new regression equations and artificial neural network in patients with diabetes

Variables included in the new regression models for estimating log GFR are log serum creatinine, and sex, age, BMI, HbA1C, and UACR on the natural scale. The relationship between log GFR and log serum creatinine was modeled as a two-slope linear spline with sex-specific knots at 0.7 mg/dL in women and 0.8 mg/dL in men. Applying multiple regression, eight new regression models were developed based on the variable above (Additional file 2: Tables S1, S2). Variables included in artificial neural network models for estimating GFR were the same as in the regression models (Table 2; Additional file 2: Table S3).

**Table 2:** The variables included in each equation and artificial neural network model

New regression equations	ANN models	Variables
New equation 1	ANN1	Age, Sex, Scr
New equation 2	ANN2	Age, Sex, Scr, HbA1c
New equation 3	ANN3	Age, Sex, Scr, BMI
New equation 4	ANN4	Age, Sex, Scr, UACR
New equation 5	ANN5	Age, Sex, Scr, HbA1c, BMI
New equation 6	ANN6	Age, Sex, Scr, HbA1c, UACR
New equation 7	ANN7	Age, Sex, Scr, BMI, UACR
New equation 8	ANN8	Age, Sex, Scr, HbA1c, UACR, BMI

*ANN* artificial neural network, *BSA* body surface area, *BMI* body mass index, *UACR* urine albumin creatinine ratio, *Scr* serum creatinine

### Determination of the optimal model

In the external validation group, biases between new GFR estimating models and the CKD-EPI equation were not significant except for the Japanese equations (Japanese equation 1 vs. CKD-EPI, −20.48 vs. 6.00 mL/min/1.73 m^2^, *P* < 0.001; Japanese eqution 2 vs. CKD-EPI, −30.67 vs. 6.00 mL/min/1.73 m^2^, *P* < 0.001). All models had improve precision (*P* < 0.001) in comparison to the CKD-EPI equation except ANN6 (*P* < 0.001). Only ANN3 had higher accuracy than the CKD-EPI equation (30 % accuracy, 88.6 vs. 80.0 %, *P* = 0.02). The Japanese equations demonstrated much lower accuracy compared with the CKD-EPI equation (Japanese equation 1 vs. CKD-EPI, 54.3 vs. 80 %, *P* < 0.001; Japanese equation 2 vs. CKD-EPI, 29.5 vs. 80 mL/min/1.73 m^2^, *P* < 0.001) (Table 3). To facilitate its clinical use, a detailed equation (Additional file 1) was developed to calculate eGFR by ANN3. A simple EXCEL software program was also created (Additional file 3).

**Table 3:** Performance of the new regression equations and ANN models in the external validation data set

	Bias (ml/min/1.73 m^2^)	Precision (ml/min/1.73 m^2^)	Accuracy (%)
CKD-EPI	−6.00	24.20	80.0
Japanese equation 1	−20.48*	22.43*	54.3*
Japanese equation 2	−30.67*	23.55*	29.5*
New equation 1	−2.49	22.50*	84.8
ANN1	−2.70	21.37*	87.6
New equation 2	−2.88	22.07*	84.8
ANN2	−4.87	20.70*	83.8
New equation 3	−3.97	21.22*	80.0
ANN3	−5.97	20.49*	88.6^‡^
New equation 4	−2.23	21.11*	84.8
ANN4	−3.08	19.96*	84.8
New equation 5	−3.97	21.46*	80.0
ANN5	−6.81	22.93*	85.7
New equation 6	−3.19	21.64*	83.8
ANN6	−5.31	25.87*	88.6
New equation 7	−2.91	21.35*	81.9
ANN7	−5.97	22.51*	87.6
New equation 8	−4.48	21.89*	81.9
ANN8	−5.73	22.30*	87.6

*CKD-EPI* *equation* Chronic Kidney Disease Epidemiology Collaboration, *ANN* Artificial neural network

* P < 0.001, ^‡^P < 0.05 comparing with the CKD-EPI equation

## Discussion

The number of people with diabetes is increasing globally, with 387 million in 2014 [23], up from 153 million in 1980 [24]. Based on a national survey carried out in 2010, the prevalence of diabetes in China was estimated to be 11.6 %, counting for 113.9 million Chinese adults with diabetes [25]. In all developed and many developing countries, diabetes has become the leading cause of chronic kidney disease [26]. It is therefore essential to accurately assess GFR in diabetic patients. Our analysis indicated ANN3 based on sex, age, serum creatinine and BMI (topological structure as 4-6-1) was the optimal model for GFR estimation in Chinese type 2 diabetic patients. In an investigation by Tsuda and colleagues, a new equation including HbA1c as a new variable was developed. However, the sample size was very small (40 cases) and the new equation had not been externally validated [9]. Further, it showed poor performance in our study.

There are two possible reasons for the superiority of ANN3 over other models. One possible reason is the introduction of BMI as a new variable, which has been explained before [9, 11, 12]. Additionally, Hsu found that BMI was related to the risk for end-stage renal disease, and GFR started to decrease when BMI ≥22.0 kg/m^2^ [27]. Kawamoto also mentioned the estimated GFR in upper normal body weight (BMI 22.0–24.9 kg/m^2)^, overweight or obese subjects (BMI ≥25 kg/m^2^) was lower than that in lower normal body weight individuals (BMI, 18.5–21.9 kg/m^2^) [28]. Another reason is the application of ANN. A multiple regression model is based on a large number of samples, and its predictive performance is restricted by the size and characteristic of the samples. ANN can simulate a relationship accurately between variables even in a small number of samples. Furthermore, multiple regression models fail to solve the high multi-collinearity between variables, whereas ANN is designed to address this problem and is flexible in modeling non-linear problems.

Two factors were assumed to influence the performance of the CKD-EPI equation in the external validation data-set. On the one hand, the development data-set of the CKD-EPI equation was quite different from that in our study. Diabetes only accounted for 29 % of the whole population in the CKD-EPI equation, while all subjects in this study were diagnosed with type 2 diabetes. There were also differences in the clinical characteristics of the two development and internal validation data-sets. The subjects of the development and internal validation data-set in our study were older than that in the CKD-EPI equation (60 ± 13 vs. 47 ± 15 y). The BMI of the development and internal validation data-set in our study were also lower than those in the CKD-EPI equation (25 ± 4 vs. 28 ± 6 kg/m^2^). The subjects of the development and internal validation data-set in our study had a higher sGFR than that in the CKD-EPI equation (81 ± 29 vs. 68 ± 40 mL/min/1.73 m^2^). On the other hand, the gold standard of our study was the ^99m^Tc-DTPA renal dynamic imaging GFR calibrated to the dual plasma sample method while the sGFR of the CKD-EPI equation was urinary clearance of ^125^I-Iothalamate. This difference in the gold standards could result in systematic bias.

Several limitations in our study should be pointed out. One of them is the relatively small number of samples in both the development and validation data-sets and the fact that subjects were restricted to a single center. Another difference is our use of ^99m^Tc-DTPA renal dynamic imaging GFR calibrated to the dual plasma sample method as the gold standard of GFR instead of inulin clearance.

## Conclusions

A new GFR estimating model (ANN3) based on sex, age, serum creatinine and BMI was selected as the optimal model for GFR estimation in Chinese patients with type 2 diabetes. However, ANN3 proved its superiority only in the external validation data-set (n = 105 patients). Additional external investigations are still required. With a larger sample size, addition of new filtration markers (Cystatin C, β2-microglobulin and β trace protein**)**, the change of GFR gold standard to inulin clearance, and the perfection of modeling machine learning methods including ANN, the predictive performance of the new models may be further improved.

## Additional files

**Additional file 1.** A detailed equation to calculate eGFR by ANN3.
**Additional file 2.** Additional Tables, Tables S1–S3.
**Additional file 3.** A simple EXCEL software to calculate eGFR by ANN3.

## Abbreviations

GFR: glomerular filtration rate
BMI: body mass index
ANN: artificial neural network
CG: Cockcroft–Gault
MDRD: modification of diet in renal disease
CKD-EPI: Chronic Kidney Disease Epidemiology Collaboration
UACR: urine albumin-to-creatinine
HbA1c: glycosylated hemoglobin
CKD: chronic kidney disease
^99m^ Tc-DTPA: technetium 99m diethylene-triaminepentaacetic acid
mGFR: measured GFR
sGFR: standard GFR
BP: back-propagation
GABP network: genetic algorithm into the BP network
SD: standard deviation
IQR: inter-quartile range
