# Supplementary material for: Development and validation of new glomerular filtration rate predicting models for Chinese patients with type 2 diabetes
Source: J Transl Med. 2015 Sep 28;13:317. doi: 10.1186/s12967-015-0674-y (PMC4591744; doi:10.1186/s12967-015-0674-y)
Supplement: Supplementary file 1 — Additional file 1. A detailed equation to calculate eGFR by ANN3. [file 12967_2015_674_MOESM1_ESM.doc]

Supplements document: New artificial neural network model

GFR=((2/(1+EXP(-2*([2*(sex-0)/(1-0)-1]*(-19.0057133717485)+[2*(age-26)/(91-26)-1]*(-8.8974464381865)+[2*(Scr*88.4-23.6)/(808-23.6)-1]*(-26.1824297975612)+[2*(bmi-16.015625)/( 38.2149901380671-16.015625)-1]*(-9.07980664700729)+(-23.8914319699347))))-1)*0.0953032927541968+(2/(1+EXP(-2*([2*(sex-0)/(1-0)-1]*(-3.20779899017519)+[2*(age-26)/(91-26)-1]*0.0855431325926812+[2*(Scr*88.4-23.6)/(808-23.6)-1]*(-15.5574442899791)+[2*(bmi-16.015625)/(38.2149901380671-16.015625)-1]*20.3605954481028+(-10.9352910454243))))-1)*(-0.0203117775096291)+(2/(1+EXP(-2*([2*(sex-0)/(1-0)-1]*23.4177306872502+[2*(age-26)/(91-26)-1]*0.334345448548183+[2*(Scr*88.4-23.6)/(808-23.6)-1]*(-35.2474703469857)+[2*(bmi-16.015625)/(38.2149901380671-16.015625)-1]*(-4.23957279507859)+(-11.0824934159376))))-1)*0.136517694328897+(2/(1+EXP(-2*([2*(sex-0)/(1-0)-1]*(-13.7750009299992)+[2*(age-26)/(91-26)-1]*10.132604085411+[2*(Scr*88.4-23.6)/(808-23.6)-1]*(-24.0067592780281)+[2*(bmi-16.015625)/( 38.2149901380671-16.015625)-1]*28.2646124174773+(-26.7239351965812))))-1)*0.00372282600597679+(2/(1+EXP(-2*([2*(sex-0)/(1-0)-1]*0.112792470241306+[2*(age-26)/(91-26)-1]*(-0.392492128226098)+[2*(Scr*88.4-23.6)/(808-23.6)-1]*(-9.25841573742427)+[2*(bmi-16.015625)/( 38.2149901380671-16.015625)-1]*0.131236741092621+(-8.05977369139342))))-1)*0.408263456970945+(2/(1+EXP(-2*([2*(sex-0)/(1-0)-1]*0.112792470241306+[2*(age-26)/(91-26)-1]*(-0.392492128226098)+[2*(Scr*88.4-23.6)/(808-23.6)-1]*(-9.25841573742427)+[2*(bmi-16.015625)/(38.2149901380671-16.015625)-1]*0.131236741092621+(-8.05977369139342))))-1)*0.0438074905870656+(-0.214997037727966)+1)*( 182.312350182068-16.3877715275376)/2+16.3877715275376

GFR, glomerular filtration rate (ml/min/1.73m^2^); Scr, serum creatinine (mg/dL); BMI, Body Mass Index (kg/m^2^)
