# Supplementary material for: Development and validation of new glomerular filtration rate predicting models for Chinese patients with type 2 diabetes
Source: J Transl Med. 2015 Sep 28;13:317. doi: 10.1186/s12967-015-0674-y (PMC4591744; doi:10.1186/s12967-015-0674-y)
Supplement: Supplementary file 2 — Additional file 2. Additional Tables, Tables S1–S3. [file 12967_2015_674_MOESM2_ESM.doc]

**SUPPLEMENTARY DADA**

Table S1 New Regression Models (New Equations 1-4)

| Equations and sex | Serum Creatinine | Equations for Estimating GFR |
| --- | --- | --- |
| New Equation1 | |  |
| Female | Scr≤0.7 mg/dl | GFR=86.9×(Scr/0.7)^-0.616^× (1.000)^age^ |
|  | Scr>0.7 mg/dl | GFR=105.0×(Scr/0.7)^-0.410^×(0.995)^age^ |
| Male | Scr≤0.8 mg/dl | GFR=106.0×(Scr/0.8)^-0.470^×(0.998)^age^ |
|  | Scr>0.8 mg/dl | GFR=132.1×(Scr/0.8)^-0.577^×(0.993)^age^ |
| New Equation2 | |  |
| Female | Scr≤0.7 mg/dl | GFR=87.7×(Scr/0.7)^-0.623^×(1.000)^age^×(0.998)^HbA1C^ |
|  | Scr>0.7 mg/dl | GFR=98.4×(Scr/0.7)^-0.404^×(0.995)^age^×(1.007)^HbA1C^ |
| Male | Scr≤0.8 mg/dl | GFR=100.0×(Scr/0.8)^-0.458^×(0.998)^age^×(1.004)^HbA1C^ |
|  | Scr>0.8 mg/dl | GFR=118.0×(Scr/0.8)^-0.561^×(0.993)^age^×(1.012) ^HbA1C^ |
| New Equation3 | |  |
| Female | Scr≤0.7 mg/dl | GFR=106.4×(Scr/0.7)^-0.597^×(1.000)^age^×(0.993)^BMI^ |
|  | Scr>0.7 mg/dl | GFR=162.6×(Scr/0.7)^-0.418^×(0.995)^age^×0.979^BMI^ |
| Male | Scr≤0.8 mg/dl | GFR=145.9×(Scr/0.8)^-0.395^×(0.998)^age^×0.989^BMI^ |
|  | Scr>0.8 mg/dl | GFR=150.0×(Scr/0.8)^-0.574^×(0.993)^age^×0.995^BMI^ |
| New Equation4 | |  |
| Female | Scr≤0.7 mg/dl | GFR=91.8×(Scr/0.7)^-0.581^×(1.000)^age^×(1.000)^UACR^ |
|  | Scr>0.7 mg/dl | GFR=89.3×(Scr/0.7)^-0.479^×(0.998)^age^×(1.000) ^UACR^ |
| Male | Scr≤0.8 mg/dl | GFR=105.2×(Scr/0.8)^-0.464^×(0.998)^age^×(1.000) ^UACR^ |
|  | Scr>0.8 mg/dl | GFR=131.8×(Scr/0.8)^-0.587^×(0.993)^age^×(1.000) ^UACR^ |

GFR, glomerular filtration rate; BSA, body surface area; BMI, Body Mass Index; UACR, urine albumin creatinine ratio; Scr, serum creatinine.

Table S2 New Regression Models (New Equations 5-8)

| Equations and sex | Serum Creatinine | Equations for Estimating GFR |
| --- | --- | --- |
| New Equation5 | | |
| Female | Scr≤0.7 mg/dl | GFR=109.1×(Scr/0.7)^-0.610^×(1.000)^age^×(0.997)^HbAIC^×(0.993)^BMI^ |
|  | Scr>0.7 mg/dl | GFR=109.1×(Scr/0.7)^-0406^×(0.995)^age^×(1.012)^HbA1C^×(0.993)^BMI^ |
| Male | Scr≤0.8 mg/dl | GFR=148.0×(Scr/0.8)^-0.396^×(0.998)^age^×(1.000)^HbA1C^×(0.989)^BMI^ |
|  | Scr>0.8 mg/dl | GFR=134.3×(Scr/0.8)^-0.558^×(0.993)^age^×(1.012)^HbA1C^×(0.989)^BMI^ |
| New Equation6 | | |
| Female | Scr≤0.7 mg/dl | GFR=91.4×(Scr/0.7)^-0.578^×(1.000)^age^×(1.000)^HbAIC^×(1.000) ^UACR^ |
|  | Scr>0.7 mg/dl | GFR=76.03×(Scr/0.7)^-0.467^×(0.998)^age^×(1.014)^HbA1C^×(1.000) ^UACR^ |
| Male | Scr≤0.8 mg/dl | GFR=99.5×(Scr/0.8)^-0.453^×(0.998)^age^×(1.005)^HbA1C^×(1.000) ^UACR^ |
|  | Scr>0.8 mg/dl | GFR=117.5×(Scr/0.8)^-0.573^×(0.993)^age^×(1.012)^HbA1C^×(1.000) ^UACR^ |
| New Equation7 | | |
| Female | Scr≤0.7 mg/dl | GFR=116.7×(Scr/0.7)^-0.556^×(1.000)^age^×(0.991)^BMI^×(1.000) ^UACR^ |
|  | Scr>0.7 mg/dl | GFR=133.4×(Scr/0.7)^-0.480^×(0.998)^age^×(0.982)^BMI^×(1.000) ^UACR^ |
| Male | Scr≤0.8 mg/dl | GFR=144.5×(Scr/0.8)^-0.389^×(0.998)^age^×(0.989)^BMI^×(1.000) ^UACR^ |
|  | Scr>0.8 mg/dl | GFR=150.7×(Scr/0.8)^-0.585^×(0.993)^age^×(0.995)^BMI^×(1.000) ^UACR^ |
| New Equation8 | | |
| Female | Scr≤0.7 mg/dl | GFR=117.2×(Scr/0.7)^-0.559^×(1.000)^age^×(0.991)^BMI^×(1.000) ^UACR^×(1.000)^HbA1C^ |
|  | Scr>0.7 mg/dl | GFR=111.7×(Scr/0.7)^-0.466^×(0.998)^age^×(0.979)^BMI^×(1.000) ^UACR^×(1.019)^HbA1C^ |
| Male | Scr≤0.8 mg/dl | GFR=146.9×(Scr/0.8)^-0.390^×(0.998)^age^×(0.989)^BMI^×(1.000) ^UACR^×(1.000)^HbA1C^ |
|  | Scr>0.8 mg/dl | GFR=134.0×(Scr/0.8)^-0.571^×(0.993)^age^×(0.995)^BMI^×(1.000) ^UACR^×(1.012)^HbA1C^ |

GFR, glomerular filtration rate; BSA, body surface area; BMI, Body Mass Index; UACR, urine albumin creatinine ratio; Scr, serum creatinine.

Table S3 Topological Structure of the ANN Models

| Models | Topological Structure | Population Size | Mean Square Error |
| --- | --- | --- | --- |
| ANN1 | 3-4-1 | 50 | 206.42 |
| ANN2 | 4-5-1 | 70 | 207.70 |
| ANN3 | 4-6-1 | 70 | 191.45 |
| ANN4 | 4-6-1 | 70 | 203.89 |
| ANN5 | 5-9-1 | 100 | 200.15 |
| ANN6 | 5-9-1 | 100 | 194.60 |
| ANN7 | 5-8-1 | 100 | 206.79 |
| ANN8 | 6-6-1 | 100 | 190.41 |

ANN, Artificial neural network;
